# Improving the classification of cardinality phenotypes using collections

**DOI:** 10.1186/s13326-023-00290-y

**Published:** 2023-08-07

**Authors:** Sarah M. Alghamdi, Robert Hoehndorf

**Affiliations:** 1 Computational Bioscience Research Center (CBRC), Computer, Electrical, and Mathematical Sciences & Engineering Division, King Abdullah University of Science and Technology, 4700 KAUST, 23955 Thuwal, Saudi Arabia; 2 King Abdul-Aziz University, Faculty of Computing and Information Technology, 25732 Rabigh, Saudi Arabia

**Keywords:** Cardinality phenotypes, Phenotype ontologies, Genotype–phenotype associations

## Abstract

**Motivation:**

Phenotypes are observable characteristics of an organism and they can be highly variable. Information about phenotypes is collected in a clinical context to characterize disease, and is also collected in model organisms and stored in model organism databases where they are used to understand gene functions. Phenotype data is also used in computational data analysis and machine learning methods to provide novel insights into disease mechanisms and support personalized diagnosis of disease. For mammalian organisms and in a clinical context, ontologies such as the Human Phenotype Ontology and the Mammalian Phenotype Ontology are widely used to formally and precisely describe phenotypes. We specifically analyze axioms pertaining to phenotypes of collections of entities within a body, and we find that some of the axioms in phenotype ontologies lead to inferences that may not accurately reflect the underlying biological phenomena.

**Results:**

We reformulate the phenotypes of collections of entities using an ontological theory of collections. By reformulating phenotypes of collections in phenotypes ontologies, we avoid potentially incorrect inferences pertaining to the cardinality of these collections. We apply our method to two phenotype ontologies and show that the reformulation not only removes some problematic inferences but also quantitatively improves biological data analysis.

## Introduction

Phenotypes are the observable characteristics of organisms and they arise from an organisms phenotype and interactions with the environment [1]. Phenotypic data is critical for deciphering the biological pathways that cause a disease [2]. A formal ontological description of phenotype data can assist in identifying, interpreting, and inferring phenotypic features from experimental data in different species [3–6]. Many ontologies cover the phenotype domain for specific organisms, such as the Human Phenotype Ontology (HP) [7] and the Mammalian Phenotype Ontology (MP) [8].

In biomedical ontologies, the Entity–Quality approach (EQ) [9] is used to logically formalize phenotypic descriptions. In the EQ approach, phenotype descriptions can be divided into (at least) two components. The first component represents the affected entity. This may include entities that are a part of an organism, including anatomical structures, liquids, and collections of entities that are part of a body. The second component describes the entity’s quality. Qualities are described in the Phenotype And Trait Ontology (PATO) [10] and qualities are divided into qualitative and quantitative qualities. For instance, the phenotype *cleft upper lip* (MP:0005170) is defined using the qualitative quality class *split* (PATO:0001786) and the entity class *upper lip* (UBERON:0001834). The phenotype class *hyperalgesia* (MP:0005407) is defined using the quantitative quality *decreased threshold* (PATO:0000708) and the entity *nociceptive behavior* (NBO:0000331). The quantitative quality *decreased threshold* (PATO:0000708) includes an implicit “normal” to which the quantity is compared [11].

The ontological representation of phenotypes has been intensively studied [11]. However, while this ontological analysis of phenotype ontologies has focused on the classification of anatomical structures and processes [12–14], the *collections* of entities that are part of a human body have not explicitly been analyzed. Phenotypes of collections include the decrease in the number, or the absence, of types of blood cells. For example, in the MP ontology, we can find a class *absent T cells* (MP:0008070) or *absent lymphocyte* (MP:0000726), as well as *decreased pancreatic alpha cell number* (MP:0009177), *increased osteocyte number* (MP:0030482).

The MP also contains a class *absent NK T cell* (MP:0008041) which is inferred to be a subclass of *absent T cell* (MP:0008070). T cells are white blood cells and have several subtypes, including natural killer (NK) T cells, regulatory T cells, and gamma-delta T cells. Consequently, the absence of NK T cell does not necessarily imply the absence of (all) T cells. More subtly, a decreased amount of a type of cell (such as NK T cell) does not imply a decreased amount of the superclass (such as T cell). Nevertheless, the MP asserts that *decreased NK T cell number* (MP:0008040) is a subclass of *decreased T cell number* (MP:0005018).

We consider this as a problem resulting from an insufficient ontological analysis of the underlying phenomenon, and offer an analysis which considers the cells of a certain cell type within a body as a collection. Based on an ontology of collections and collectives [15], we reformulate the axioms pertaining to classes using the *amount* (PATO:0000070) quality in phenotypes ontologies. Specifically, the MP ontology contains 569 phenotype classes related to the cardinality of a collection of cell types within a body and the HP ontology contains 63 cardinality phenotype classes related to cells contained in a body. We apply the results of our analysis to the MP and HP, modify the axioms of abnormalities of collections of cells, and reclassify the ontology to derive a novel taxonomy of phenotype classes. We then use this novel taxonomy with a semantic similarity measure to predict gene–disease associations based on phenotypic similarity between genes annotated by MP and disease annotated by HP. We find that our new formulation of cardinality phenotypes improves predictions of gene–disease associations based on phenotypic similarity.

## Materials and methods

### Entity–Quality statements of collections

Phenotypes include the organism’s appearance, development, and behavior. The phenotype of an organism is determined by its genotype as well as its interactions with the environment [16]. In biomedical ontologies, phenotypes are represented using the EQ formalism [9, 12]. The EQ formalism splits a phenotype into two parts, the Entity (E) which is a class from an ontology that contains parts of an organism (such as anatomy or cell types), and a Quality (Q) from the PATO [10]. The common formal representation of phenotype classes using Description Logic syntax [17] is either

1 $$\begin{aligned} EQ \sqsubseteq \exists has\_part.(Q \sqcap \exists characteristic\_of.E ) \end{aligned}$$

or

2 $$\begin{aligned} EQ \sqsubseteq \exists has\_part.(E \sqcap \exists has\_characteristic.Q ) \end{aligned}$$

$\sqsubseteq$ represents the subsumption or subclass axiom, $\exists R.C$ represents the existential restriction of relation *R* of class *C*, and $\sqcap$ represents conjunction. Here, we exclusively use the first formulation. This formulation allows for describing an entity with some qualitative or quantitative quality. For instance, the phenotype *decreased vertebrae number* (MP:0004645) is defined using the quality class *decreased amount* (PATO:0001997) and the entity class *vertebra* (UBERON:0002412). In Description Logic, the corresponding axiom is:

3 $$\begin{aligned} \text {`decreased vertebrae number'} \equiv \exists has\_part. (\text {`decreased amount'} \sqcap \nonumber \\ \exists characteristic\_of. \text {vertebra} \sqcap (\exists has\_modifier. \text {abnormal}) ) \end{aligned}$$

where $\equiv$ represents equivalence.

**Fig. 1:**
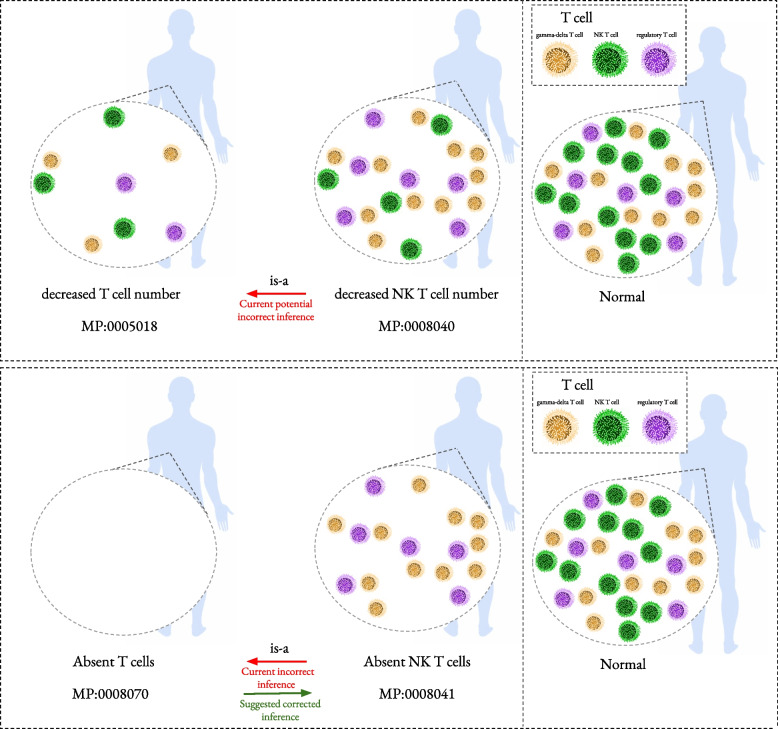
This figure presents examples of a potentially incorrect inference between phenotypes. At the top is the axiom inferred between (decreased number of NK T Cells) and the phenotype (decreased number of T Cells), at the bottom is the axiom inferred between (absent NK T Cells) and the phenotype (absent T Cells) as well as the suggested revised classification

Similar EQ axiom patterns are utilized in many phenotype ontologies including the HP and the MP ontology. However, the use of qualities that express an increased or decreased amounts may lead to inferences that could be considered to be incorrect. In Fig. 1, we illustrate some of the consequences of the current axiom patterns. In the first example, the axioms that define the classes *decreased T cell number* and *decreased NK T cell number* are:

4 $$\begin{aligned} \text {`decreased T cell number'}\equiv & {} \exists has\_part. (\text {`decreased amount'}\nonumber \\&\sqcap&\exists characteristic\_of.\text {`T cell'} \sqcap (\exists has\_modifier. abnormal) ) \end{aligned}$$

5 $$\begin{aligned} \text {decreased NK T cell number}\equiv & {} \exists has\_part. (\text {`decreased amount'} \nonumber \\&\sqcap&\exists characteristic\_of.\text {mature NK T cell} \sqcap (\exists has\_modifier. \text {abnormal}) ) \end{aligned}$$

As a consequence of these axioms and the fact that every *NK T cell* is a *T cell*, we can infer that *decreased NK T cell number* is a subclass of *decreased T cell number*. *decreased NK T cell number* is a class representing the phenotype of having an abnormally low number of NK T cells; *decreased T cell number* is a class representing the phenotype of having an abnormally low number of T cells in the blood. Considering the intended meaning of these classes, and the way in which they are used in databases of phenotypes, the inferred subclass statement is likely incorrect and not intended; it is not necessarily the case that the decreased number of NK T cells in the blood results in a decreased number of T cells in the blood (because other types of T cells may increase as a consequence of a reduced NK T cell count).

In the second example (bottom of Fig. 1), we illustrate another example that may be even more explicit in how the axioms can lead to consequences that contradict the intended meaning of the classes; we illustrate the relation between the classes *absent NK T cells* (MP:0008041) and *absent T cells* (MP:0008070), which are formally defined as:

6 $$\begin{aligned} \text {`absent NK T cells'}\equiv & {} \exists has\_part. (\text {absent}\nonumber \\&\sqcap&\exists characteristic\_of.\text {`mature NK T cell'} ) \sqcap (\exists has\_modifier. abnormal)) \end{aligned}$$

7 $$\begin{aligned} \text {`absent T cells'}\equiv & {} \exists has\_part. (\text {absent}\nonumber \\&\sqcap&\exists characteristic\_of.\text {T cell} ) \sqcap (\exists has\_modifier. \text {abnormal})) \end{aligned}$$

Again, based on these axioms, it can be inferred that *absent NK T cells* is a subclass of an *absent T cells*, which is clearly not the case. Instead, the opposite should be true [13, 14]: if there are no T cells within a body, this will imply that there are no NK T cells in a body (because NK T cells are special types of T cells).

### Ontologies and datasets

For our experiments, we used the MP [18] (04-11-2021 release), and the HP [7] (10-10-2021 release). For the purpose of providing a quantitative evaluation, we acquired human gene–disease associations from the Mouse Genome Informatics (MGI) database [19] which are based on those from the Online Inheritance in Men (OMIM) database [20] and other sources, including NCBI’s Gene Review [21]. This dataset includes 4,930 human genes, 4,619 OMIM diseases, and 17,833 human gene–disease associations. Among those, 425 diseases have at least one cardinality phenotype with 873 gene–disease associations. We downloaded this data in March 2023 from the MGI (file MGI_DO.rpt). To annotate the human genes, we use the phenotypes of their mouse orthologs. This information we acquired from MGI from the file HMD_HumanPhenotype.rpt. The version we used was downloaded in March 2023. Human disease–phenotype annotations were obtained from the HP database [22], from the file phenotype_to_gene.txt downloaded in March 2023. Mouse gene–phenotype annotations were obtained from MGI database MGI_GenePheno.rpt which uses MP, downloaded in March 2023.

### Integrating HP and MP with corrected cardinality phenotypes

**Fig. 2:**
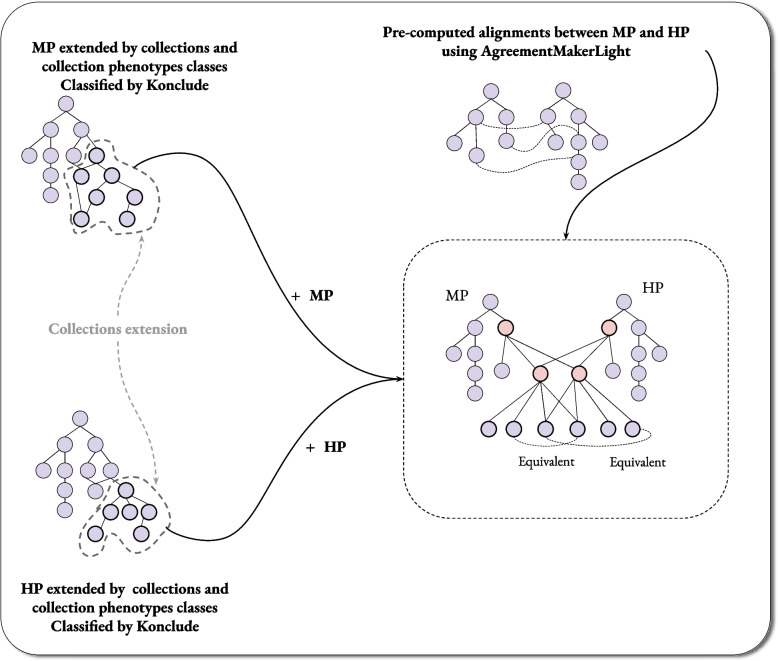
This figure explains the processes we followed to integrate MP, HP, and the expanded collection classes. We applied this update to MP and HP separately before classifying both ontologies using the Konklude reasoner. We generated a merged ontology using these additional axioms, MP and HP, and pre-computed equivalence alignments of MP and HP

To evaluate our new representation of cardinality phenotypes, we integrated HP and MP, extended by reformulating cardinality phenotypes with our proposed representations, as described in Representing phenotypes of collections section. We created 211 collection classes, 634 phenotypes of collection classes, and 214 grouping classes. Initially, we extended the MP ontology and the HP ontology independently, while maintaining the same identifiers for the grouping classes and the collection of cell classes. Then, we categorized both extended ontologies using the Konclude reasoner [23]. We apply the Konklude reasoner as it supports OWL 2 DL and the axioms we have defined for collections and collection phenotypes include negation and universal restrictions. We combine MP, HP, and the (deductively closed) extension with collections. Then, we add equivalent class axioms between the MP and HP classes using the AgreementMakerLight ontology alignment tool [24]. Our approach is illustrated in Fig. 2.

### Semantic similarity

We utilized Resnik’s semantic similarity [25] to compare a set of phenotype classes representing genes and diseases. Resnik’s similarity is a similarity measure based on information content (IC). IC is a class specificity measure [26] and is defined as:

8 $$\begin{aligned} IC(class)=-log(p(class)) \end{aligned}$$

where *p* represents the probability of a class being used to annotate an entity (gene or disease). The similarity between two ontology classes is defined as the information content of the most informative common ancestor (MICA) of two classes:

9 $$\begin{aligned} sim_{Resnik}(g_i,d_j)=IC(MICA(g_i,d_j)) \end{aligned}$$

where $g_i$ is defined as the $i^{th}$ phenotype annotation of gene *g* and $d_j$ is the $j^{th}$ annotation of disease *d*. As we compare groups of classes, we use the best match average method (BMA) [27] to calculate the similarity between genes and diseases:

10 $$\begin{aligned}{} & {} sim_{BMA}(gene,disease)=\end{aligned}$$

11 $$\begin{aligned}{} & {} \dfrac{\sum _{i=1}^{g_n}\max _{1 \le j \le d_n}(sim_{Resnik}(g_i,d_j))}{2*g_n} + \dfrac{\sum _{i=1}^{d_n}\max _{1 \le j \le g_n}(sim_{Resnik}(d_i,g_j)) }{2*d_n} \end{aligned}$$

### Implementation

We developed our software in Groovy [28] using the OWLAPI [29] to generate ontology axioms and using the Semantic Measures Library (SML) [30] to compute semantic similarities. We used the Numpy library [31], scikit-learn [32] for evaluation, and Protégé [33] to visualize the ontology and classes.

## Results

### Ontological analysis of collections and maximal collections

Our aim is to find a formal ontological representation of phenotypes of collections of cells (and other entities) that is as close as possible to the EQ formalism used in the phenotype ontologies yet avoids the problematic inferences we identified. To achieve this goal, we reuse an ontological theory of collections and collectives [15] which introduces different properties of collections and collectives. Here, we are primarily concerned with defining collections of entities (such as cells) that are either contained in or part of an organism.

We focus on biomedical applications for our formulation where we are interested in collections of entities that are part of a body. As such, members of a collection can change over time, and collections can be empty (such as in the case of absent T cells). Empty collections are important as they are used to signify disorders, such as those resulting from congenital abnormalities, where certain types of cells or types of chemicals cannot be produced and therefore these collections are empty.

We can define mereological relations between collections [34]. Of particular importance for us is the relation between subclass (of members) and the parthood between the respective collections. For example, every T cell is a kind of lymphocyte; consequently, while a collection of T cells is a kind of collection of lymphocytes, it is also the case that every collection of T cells is a *part of* a collection of lymphocytes.

This may just be trivially true due to the reflexivity of *part-of* as long as we do not restrict these collections further. However, we are not particularly interested in just defining collections of types of cells; there are many collections of T cells that are part of a human body. Instead, we are interested in the notion of a “maximal” collection of entities that are a part of a body, i.e., the collection of *all* entities of type *X* that are a part of a single (instance of a) body. We call this the maximal collection of *X* within a *Y* (where *Y* is a class representing an organism or the body of an organism). We can define this class in first order logic (where $\leftrightarrow$ represents bi-conditional logical symbol, read as “if and only if”, $\wedge$ is a conjunction, $\exists$ and $\forall$ are the existential and universal quantifiers):

12 $$\begin{aligned} \text {X-Collection}(x) \leftrightarrow (\exists y( Y(y) \wedge ( \forall a ( X(a) \wedge part\_of(a,y) ) \leftrightarrow has\_member(a,x)))) \end{aligned}$$

or, using temporalized parthood and membership relations (such as used in the Basic Formal Ontology, BFO [35]):

13 $$\begin{aligned} \text {X-Collection}(x,t) \leftrightarrow (\exists y( Y(y) \wedge ( \forall a ( X(a) \wedge part\_of(a,y,t) ) \leftrightarrow has\_member(a,x,t)))) \end{aligned}$$

We cannot equivalently represent these axioms in a Description Logic that is used to represent phenotype ontologies. However, we may be able to assume that the universe over which we quantify ranges only over entities that are a part of a single body, allowing us to omit the condition on the right-hand side of Eqn. 12. We then define $\text {X-Collection}$ as the collection of all the individuals of type *X* (where “all” ranges over parts of *Y*, e.g., the parts of a body):

14 $$\begin{aligned} \text {X-Collection}(xc) \leftrightarrow (\forall a ( X(a) \leftrightarrow has\_member(a, xc))) \end{aligned}$$

While this is an axiom in first order logic, we are mainly interested in an implementation in a Description Logic such as the one underlying OWL 2 DL [36] so that our results are compatible with the MP and HP. In Description Logic, we assert two axioms for these collection classes containing *X*:

15 $$\begin{aligned} \text {X-Collection} \sqsubseteq \forall has\_member. X \end{aligned}$$

16 $$\begin{aligned} X \sqsubseteq \exists member\_of. \text {X-Collection} \end{aligned}$$

These axioms do not yet capture the intuition that an $\text {X-Collection}$ should be the collection of all *X* in the domain of discourse; we can further strengthen these axioms by asserting that there is only one such collection:

17 $$\begin{aligned} \text {X-Collection} \equiv \{ \text {x-collection} \} \end{aligned}$$

Here, $\text {x-collection}$ is a new individual name that is not used anywhere else, and $\{ ... \}$ is the Description Logic constructor for nominals (class descriptions defined by enumerating the class members). Because every instance of *X* will be a member of this collection (Eqn. 16), *X* will approximate the notion of the maximal collection of *X*s within a body.

Nevertheless, this is only a weak approximation of the first order logic axiom. In particular, we can infer from the first order logic axioms that, if *X* is a subclass of *Y*, then every $X\_Collection$ is a part of some $Y\_Collection$. In Description Logic, this is not inferred and we instead assert this consequence directly as a set of axioms: given an ontology *O* and its deductive closure $O^\vdash$, and for every pair *X* and *Y* such that $X \sqsubseteq Y \in O^\vdash$, we assert $\text {X-Collection} \sqsubseteq \exists part\_of. \text {Y-Collection}$.

### Representing phenotypes of collections

Our aim is to identify a set of axioms for representing quantitative phenotypes (phenotypes of collections) so that the inferences drawn from the axioms more accurately reflect the intended inferences from these axioms, while we aim to preserve interoperability with other axioms in phenotype ontologies that do not pertain to collections; consequently, we still have to follow the EQ formalism and the way it is implemented in phenotypes ontologies.

#### Qualities of cells and qualities of collections

We will use the following terms to refine the formal characterization of cardinality phenotypes in phenotype ontologies:

- *X* and *Y* are classes from an anatomy or cell type ontology, such as the class *T cell* or *NK T cell*;
- $\text {X-Collection}$ and $\text {Y-Collection}$ are classes representing (maximal) collections where all the members of these collections are instances of X and Y, respectively.
- *amount* is a quality (including the class *amount* (PATO:0000070), *increased amount* (PATO:0000470), *decreased amount*) (PATO:0001997), *absent* (PATO:0000462), and *duplicated* (PATO:0001473) defined in the PATO ontology.

The current phenotype ontologies represents phenotypes of collections in the EQ formalism where the Entity *E* is a cell class and the quality *Q* is a phenotype class from PATO (Eqn. 1); the class from PATO will be a subclass of the quality *quantitative* in PATO, such as *amount*. We reformulate these phenotype classes using the collection classes we defined earlier. We define a *CP* class that represents a cardinality phenotype on a collection of cells, employing an EQ pattern where the entity is the collection of cells, $\text {X-Collection}$, defined as follows:

18 $$\begin{aligned} CP \sqsubseteq \exists has\_part.( amount \sqcap (\exists characteristic\_of. \text {X-Collection}) \sqcap \nonumber \\ (\exists has\_modifier. abnormal)) \end{aligned}$$

Specifically, for a phenotype of the collection of T cells, we first define the class $\text {T cell-Collection}$ and then an *Abnormality of T cell number* as:

19 $$\begin{aligned} \text {`Abnormality of T cell number'} \sqsubseteq \exists has\_part.( amount \sqcap \nonumber \\ (\exists characteristic\_of. \text {`T cell-Collection'})\sqcap (\exists has\_modifier. abnormal)) \end{aligned}$$

Another type of cardinality abnormality is the absence of certain entity *X*. These absence phenotypes are currently formulated using the same *EQ* patterns, with *Q* being the class *absence* (PATO:0000462), therefore leading to the consequence that an absence of NK T cells is a subclass of an absence of T cells. We can use the notion of the empty collection to formulate absence:

20 $$\begin{aligned} absent\_X \equiv \exists has\_part.(quality \sqcap \exists characteristic\_of.\nonumber \\ (X\_Collection \sqcap \forall has\_member. \bot ) \sqcap (\exists has\_modifier.abnormal)) \end{aligned}$$

Here, $\bot$ represents the bottom concept interpreted as an empty set. While we can use this notion of an empty collection, we still have to establish a relation between the empty collection of *X* and a body not having any instance of *X* as part; this would be possible in first order logic but not easy in Description Logic. Consequently, we also use the following formulation to relate absence to the parthood relation (where $\lnot$ represents negation):

21 $$\begin{aligned} absent\_X \equiv \lnot \exists has\_part. (quality \sqcap \exists characteristic\_of. X) \end{aligned}$$

By defining $absent\_X$ twice we also make the right-hand sides of the definitions equivalent and thereby can infer that having a quality of an empty collection of *X* is equivalent to not having a quality of *X*, i.e., we ensure equivalence between the two distinct formulations of absence.

We further define grouping classes, based on collections and based on qualities. For instance, any cardinality abnormality, whether it is a decrease or increase in number of T cells can be classified as a cardinality abnormality of collection of T cells. Therefore, we create the class *CXP* to group all the abnormalities of a certain collection *XCollection* defined as follows:

22 $$\begin{aligned} CXP \sqsubseteq \exists has\_part. (quality \sqcap \exists characteristic\_of. X\_Collection\sqcap \nonumber \\ \exists has\_modifier. abnormal) \end{aligned}$$

Another way to classify cardinality phenotypes is to group them based on qualities. For instance, we create a class that groups all the “increased cardinality” phenotypes. Therefore, we create the class *CQ* to group abnormalities of type *amount* of any collection *XCollection* using the root collection class *C*. *CQ* is defined as follows:

23 $$\begin{aligned} CQ \sqsubseteq \exists has\_part. (Q \sqcap \exists characteristic\_of.C \sqcap \exists has\_modifier. abnormal) \end{aligned}$$

**Fig. 3:**
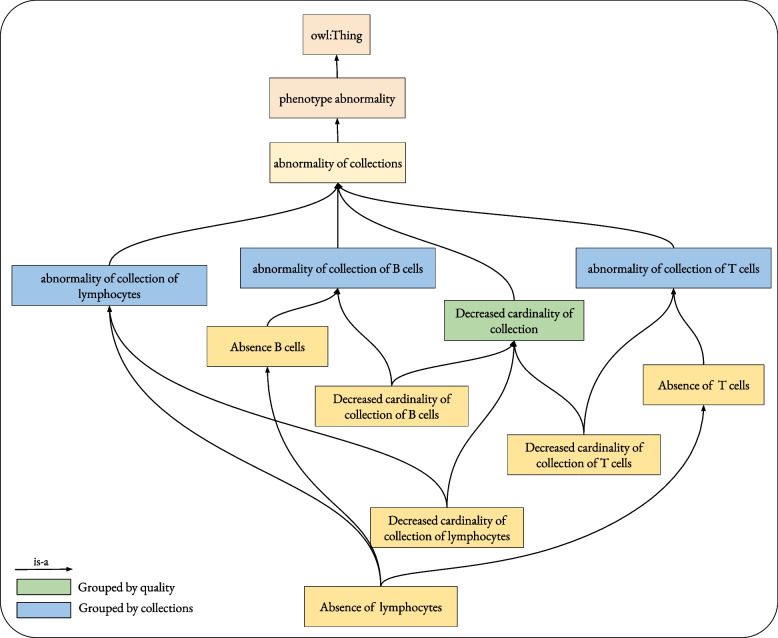
Illustration of grouping classes; the green class is an example of a quality-based grouping class *decreased cardinality*. This class will be inferred to be the superclass of every abnormality of a decreased cardinality of any collection of cells, including *decreased cardinality of B cells*, *decreased cardinality of T cells*, *decreased cardinality of lymphocytes*, etc. the blue classes are examples of grouping based on the entities *collection of T cell*, *collection of B cell*, and *collection of lymphocytes*. For instance, the class *abnormality of collection of T cell* will be inferred to be the superclass of any abnormality of *collection of T cells*, including *decreased cardinality of T cells*, and *absent T cells*

Figure 3 illustrates the use of these grouping classes.

### A revised hierarchy of cardinality phenotypes improves prediction of genes associated with rare disease

**Fig. 4:**
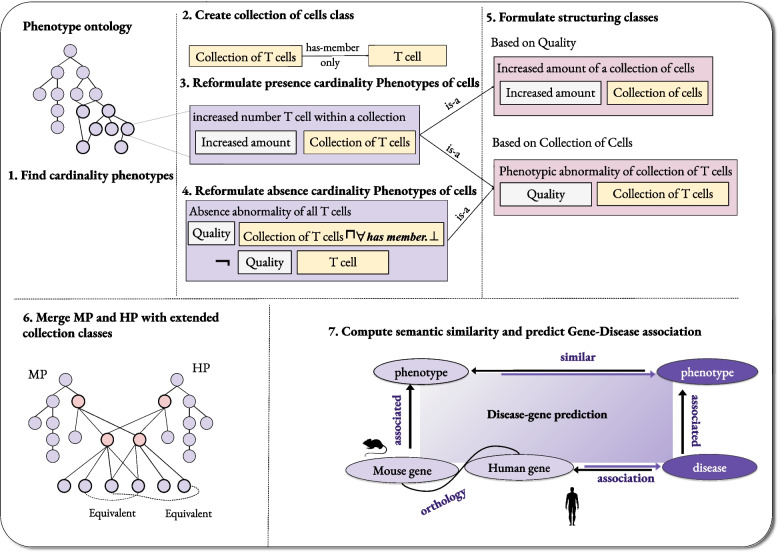
This figure present the workflow of this work with the example of the phenotype (increased number of T Cells) and the phenotype (absent T Cells). In this particular example, we created the class (collection of T cells) representing all the T cells. Then we created the phenotype classes (increased number of T Cell within a collection) and (absence of all T Cells). We added two structuring classes, one based on the quality (increased amount) and one based on the collection (collection of T cell). In order to evaluate, we applied a quantitative evaluation based on a biomedical task, in particular the gene–disease association prediction task using semantic similarity between phenotypes

We quantitatively evaluate the new classified phenotype ontologies based on our new formulation of cardinality phenotype. The approach we use follows a task-based evaluation [37, 38]. In a task-based evaluation, we apply different variants of an ontology and evaluate their performance with respect to a specific task. We utilize an ontology-based phenotypic similarity measure to predict the association between genes and diseases. For this experiment, we utilized a dataset from the Mouse Genome Informatics (MGI) database [21] which includes associations between human genes and Mendelian diseases as reported in OMIM database. Using phenotypes associated with mouse orthologs of human genes (from MGI) and human disease phenotypes from the HP database [7], we calculate the degree of similarity between their phenotypes, rank genes for each disease, and determine whether we can identify the correct disease-associated gene at a certain rank; we quantify the performance using the area under the receiver operating characteristic (ROC) curve [39], similar to other studies [3, 4]. To directly compare human and mouse phenotypes, we use an integrated ontology consisting of HP and MP, where equivalences between HP and MP classes have been determined using an automated ontology alignment tool (see Integrating HP and MP with corrected cardinality phenotypes section). The use of ontology alignment is in contrast to an integration based on axioms as used in the integrated Monarch knowledge graph [40] or the PhenomeNET ontology [4]; while the integrated ontologies may provide more alignments between classes, relying exclusively on ontology alignment allows us to evaluate the modifications to HP and MP directly without the need to rewrite or add further axioms. Figure 4 illustrates the steps of our evaluation.

We compare only diseases and genes which are annotated with at least one cardinality phenotype, using 425 diseases and 4,471 genes. We first compare their phenotype similarity only based on cardinality phenotype classes, i.e., ignoring all other phenotypes; we compare their similarity twice: first we use the original classification of phenotype classes in HP and MP, and, second, we use the revised classification of the cardinality phenotype classes based on our ontological analysis. For each disease, we rank all genes based on their similarity (the gene with the highest phenotype similarity is ranked first), and evaluate where we rank the correct disease-associated gene using the area under the ROC curve (ROCAUC).

Using the original classification of cardinality phenotypes in HP and MP, we obtain a ROCAUC of 0.6931 whereas the ROCAUC increases to 0.7384 with our revised hierarchy. While this demonstrates an improvement, it is not a realistic scenario in finding gene–disease associations because the majority of phenotypes is omitted. As a second test, we compared the same set of genes and diseases using all their phenotype annotations (cardinality phenotypes and non-cardinality phenotypes). Again, the ROCAUC improves from 0.9166 with the original classification of phenotypes to 0.9265 with the revised classification.

## Discussion

We provide an ontological analysis of the question what constitutes an abnormality of a collection; is an abnormality the absence of a normal member of a collection or the presence of a single abnormal member? In phenotype ontologies such as MP or HP, collections are not explicitly introduced. Furthermore, collections are not explicitly available in anatomy ontologies; while Uberon [41] contains classes such as *Collection of hairs* (UBERON:0010164), it does not contain collections of cells.

There has been a substantial body of work on defining absence of entities in phenotype ontologies [13, 14]. However, the majority of this research has also focused on the absence of single anatomical entities or processes, not on the absence of members of collections. Our analysis, building on an established theory of collections and collectives [15], fills this gap. We also provide axioms in first order logic and an approximation in Description Logic that leads to desirable entailments.

Importantly, we are able to evaluate our ontological treatment of abnormalities of collections both qualitatively (through automated reasoning) and quantitatively through a task-based evaluation. Phenotype ontologies are widely used in finding gene–disease associations or ranking and prioritizing variants in rare disease [3, 42–47]. Our task-based evaluation demonstrates how our work is directly relevant to these kind of applications and how refinement of ontologies can improve the application of phenotype ontologies for personalized interpretation of genomic variants.

One specific example where we improve the prediction of gene–disease associations is for the disease *Omenn syndrome* (OMIM:603554) which is associated with three genes: DCLRE1C (ENTREZ:64421), RAG2 (ENTREZ:5897) and RAG1 (ENTREZ:5896). Originally, using phenotypic similarity based on MP and HP, the first correct gene associated with Omenn syndrome was found at rank 409; with the improved phenotype representation, the highest-ranked disease-associated gene was found at rank 11. Among the annotations of this disease, we found the class *Severe B lymphocytopenia* (HP:0005365), i.e., absent B cells. In the semantic similarity computation, the information content of this class changed from originally 6.2645 bits to 10.8289 with the use of collection phenotypes. Similarly, among the classes that are used to annotate all of the three genes associated with this disease, we find the class *absent B cell* (MP:0008071) which originally had an information content of 6.0927 bits and increased to 7.1653 using the new ontology formulation of a collection of classes.

The application of collections to represent cardinality phenotypes extends beyond the cardinality of collections of cells. Similar issues as those we identified for collections of cells can be found, for instance, for the cardinalities of collections of chemicals. However, for chemicals, the entity in which they are contained (or rather, the entity with respect to which average numbers are counted) may not be “body” but rather certain cell types within a body. For example, the phenotypes *Increased level of galactonate in red blood cells* (HP:0410063) or *Increased level of N-acetylneuraminic acid in fibroblasts* (HP:0410157) or *Storage in hepatocytes* (HP:0031137) represents the increase of accumulated material in specific cell types, not within the entire body. Here, a refined ontological analysis may “stack” collections, i.e., define collections of chemicals within members of collections of cell types. However, we leave this analysis for future work.

## Conclusion

We have identified axioms that cause undesirable inferences in several phenotype ontologies. These axioms relate to cardinality phenotypes, i.e., quantitative phenotypes related to the *amount* (PATO:0000070) quality. We have provided a novel ontological analysis of these phenotypes based on an ontological theory of collections; our analysis allowed us to reformulate a large number of classes in phenotype ontologies, and reclassify the ontology in order to derive a new taxonomy of phenotype classes. We demonstrated that this novel classification can improve the use of ontologies in biomedical tasks.

## Data Availability

All software developed for this project and necessary information to reproduce results is available at https://github.com/bio-ontology-research-group/CardinalityPhenotypes.
